# Methylphenidate treatment of adult ADHD patients improves the degree of ADHD severity under routine conditions

**DOI:** 10.1007/s00702-020-02226-7

**Published:** 2020-09-03

**Authors:** Wolfgang Retz, Michael Rösler, Roland Fischer, Claudia Ose, Richard Ammer

**Affiliations:** 1 Klinik für Psychiatrie und Psychotherapie, Universitätsmedizin Mainz, Mainz, Germany; 2 Institut für Gerichtliche Psychologie und Psychiatrie, Universitätsklinikum des Saarlandes, Kirrberger Straße, 66421 Homburg/Saar, Germany; 3 MEDICE Arzneimittel Pütter GmbH & Co. KG, Iserlohn, Germany; 4 Institut für Medizinische Informatik, Zentrum für Klinische Studien Essen, Universitätsklinikum Essen, Biometrie und Epidemiologie, Essen, Germany; 5 Klinik für Innere Medizin, Universitätsklinikum Muenster, Muenster, Germany

**Keywords:** Methylphenidate, ADHD therapy, Dosage, Efficacy, Tolerability, Symptoms relief

## Abstract

Attention-deficit hyperactivity disorder (ADHD) is associated with substantial personal and social impairments. Besides psychosocial interventions, current guidelines recommend a therapy with methylphenidate (MPH). This prospective, non-interventional study aims to investigate the efficacy and tolerability of MPH treatment of adult ADHD patients in a real-world setting. 468 adult patients with newly diagnosed ADHD were observed for 12–14 weeks. Primary efficacy endpoint was the clinical global impression (CGI) by the physician. Secondary endpoints comprise patient evaluation (Wender–Reimherr self-report, WR-SR), safety, tolerability, and dosage of MPH. With a mean daily dose of 35.8 (±17.0) mg MPH, the population of patients being severely/most extremely ill or markedly ill decreased by 64% and 61%, respectively. According to physicians’ assessment (CGI), 74.5% of patients were identified as treatment responders. The total score of patient-based assessment (WR-SR) improved by 23.5% (50.1 ± 40.3 points) with the most profound improvement in attention deficit (−30.0%), disorganization (−26.6%), and hyperactivity / unrest (−23.3%). Self-evaluation revealed a responder rate of 35.4%. In summary, MPH treatment improves the degree of ADHD severity under routine conditions. In addition, activities of daily living were facilitated when taking MPH. The rather poor responder rates determined by patient assessment as well as the comparatively low applied mean daily dose of 35.8 mg (median 40 mg) indicate sub-optimal dosing under routine conditions, not exploiting the full beneficial therapeutic potential of MPH.

## Introduction

Attention-deficit hyperactivity disorder (ADHD) is a mental disorder associated with various personal and social impairments (Kabisch et al. 2011) and represents the most prevalent disorder found in childhood and adolescence with a prevalence of 4–7% (Spencer et al. 2007), which persists into adulthood in 65% of cases (Faraone et al. 2006). In Germany, up to 4.7% of adults suffer from ADHD (Ebert et al. 2003) with typical symptoms such as inattention, disorganization, impulsivity, agitation, distractibility, mood fluctuations, and poor planning capabilities (Faraone et al. 2006; Erskine et al. 2016; Fields et al. 2017). For adequate treatment, current guidelines recommend a multi-modal approach including psycho-social and pharmaco-therapeutic interventions (BfArM 2003; Medice 2017). In pharmacotherapy, MPH is recommended as first (BfArM 2003; S3 2018) or co-first line medication (NICE 2018) as its efficacy has been proven not only in children, but also in adults with ADHD in many randomized, placebo-controlled studies (RCTs) (Bottelier et al. 2017; Chobanian et al. 2003; Kessler et al. 2007; Wender et al. 1985; Rösler et al. 2009, 2013, 2005; Spencer et al. 1995; Retz et al. 2012).

RCTs with a high level of evidence and a low risk for systematic flaws represent the gold standard for evaluating efficacy and tolerability of a drug (Harbour and Miller 2001). Precise criteria for inclusion and exclusion of patients assure a relatively homogenous study population of patients recruited with comparable patient characteristics, demographic data, burden of disease, co-morbidities, and co-medication (Adler 2008), but frequently question the transferability of study results to clinical routine practice.

Non-interventional studies (NIS), in contrast, investigate efficacy and safety of a drug in a very heterogeneous group of patients and allow judgment under real conditions seen in routine clinical practice (Röhrig et al. 2009). With a usually large number of patients included, also less common adverse events can be documented. So far, there are only limited data for MPH in adults with ADHD collected from non-interventional studies. Thus, the aim of this NIS was to gather further insights in the use of MPH and its efficacy and tolerability of MPH in adults with newly diagnosed ADHD. Medikinet® adult was the first MPH product in Germany approved for treating adults with ADHD, with a modified release profile and investigated in RCTs EMMA and QUMEA (Retz et al. 2012; Rösler et al. 2009).

In the presented multi-center cohort study, we investigate a large, heterogeneous set of patients and the effect of MPH on both ADHD symptoms (hyperactivity, impulsivity, inattention) and emotional factors such as coping with stress and mood instabilities.

## Patients and methods

### Study design

We present a prospective, non-interventional study according to §4,23(3) German drug law (AMG) supported by a positive vote by the ethics committee Saarland. Patients included had a newly diagnosed ADHD and were eligible for pharmacotherapy with methylphenidate (MPH) based on the assessment of the treating physician. Diagnosis of ADHD according to DSM-IV was validated by applying the screening test with self-evaluation ASRS-V1.1 and a standardized interview IDA (integrated diagnosis of ADHD in adults) (Katzman et al. 2017; Retz et al. 2013).

Pharmacotherapy with MPH was in line with the approved label (Medice 2017) stressing careful dose titration. Duration of observation was 12–14 weeks (mean 3.3 ± 1.6 months) with an initial examination at the day of inclusion (visit 1) and a final examination at the end (visit 2). Parameters recorded were vital signs, extend of ADHD core symptoms and ADHD associated symptoms such as emotional dysregulation, level of disorganization, and social adaptiveness by applying Wender–Reimherr self-Evaluation (WR-SB, visit 1 and 2). Additionally, data on history, diagnosis and therapy (visit 1) as well as change in clinical global impression (CGI), dosing, tolerability (visit 2) were documented.

Primary endpoint was efficacy-based physician’s assessment CGI. Secondary endpoint was patient self-evaluation (WR-SB) as well as tolerability and dosage of MPH.

### Efficacy assessment

Efficacy of treatment with MPH was assessed by means of CGI measures, a 7-step scale for severity of symptoms (CGI-S, 1 = no disorder, 7 = severe disorder) and a 7-step scale for improvement of symptoms (CGI-I, 1 = impressive improvement, 4 = no improvement, 7 = strong deterioration).

Efficacy was also measured by patient self-assessment using Wender–Reimherr self-evaluation (WR-SB) which applies a five-step scale both for classical criteria inattention, hyperactivity, impulsivity and additional symptoms typical for adults such as disorganization, emotional dysregulation and problems in social adaptiveness. The scale comprises 53 items addressing 10 categories. All items need to be assessed in a 5-step Likert scale (1 = does not apply to me at all; 5 = applies to me very well) resulting in values between 53 (low burden of disorder) to 265 points (high burden). Responders were defined as patients experiencing a very good to good improvement in CGI (CGI-I < 2) or reduction in WR-SB by > 30%.

### Safety assessment

Endpoints for safety were vital parameters and overall tolerability. Vital signs (blood pressure, heart rate, weight, BMI, and appetite) were documented in both visits and after changes in dosing of MPH. In visit 1, additional characteristics (age, sex, type of ADHD, co-morbidities, co-medication and initiation of MPH treatment) were recorded. Over the course of the study, related and non-related adverse events and suspected events were documented and reported if severe.

#### Statistics

Descriptive statistics comprises absolute, relative and adjusted relative frequencies, multiple recordings, cross tables, means, standard deviations, median, and range. Wilcoxon rank-test was applied for calculating changes in CGI overall and sub-scores, WR-SB using software SAS version 9.4 running on a Windows 7TM personal computer. Co-medication was coded according to WHO-ATC. Coding of adverse events and co-morbidities followed MedDRA version 17.0.

## Results

### Patient characteristics

468 adult ADHD patients from 126 sites (mean 3.8 ± 4.0 patients per site, range 2–7 patients per site) were followed for 3.3 ± 0.5 months in average. The age of patients (57.9% male) ranged from 18 to 71 years with a mean of 32.4 ± 10.8 years.

The type of ADHD, was most frequently classified as combined type in 209 patients, 44.7%; inattentive type in 162 patients (34.6%) and hyperactive-impulsive type in 43 patients (9.2%). Co-morbidities were found in 246 patients, 52.6% of the population. Frequently, patients suffered from psychiatric disorders (197 patients, 42.1%) with depression the lead co-morbidity (129 patients, 27.6%) (Table 1).

**Table 1:** Patient characteristics

	Number of patients	
	Absolute	In %
Sex (*n* = 468)		
Male	271	57.91
Female	197	42.09
Age (*n* = 462)		
Mean	32.49 years	
Min–Max	18–71 years	
Weight (*n* = 460)		
Mean	79.8 kg	
Min–Max	54–97 kg	
BMI (*n* = 460)		
Mean	24.95 kg/m^2^	
Diagnosis (IDA, *n* = 468)		
ADHS in childhood	11	2.35
ADHD, combined type	209	44.66
ADHD, inattentive type	162	34.62
ADHD, hyperactive-impulsive type	43	9.19
No diagnosis	43	9.19
Co-morbidities (*n* = 468, incidence > 1.9%)		
Total	246	52.56
Depression	129	27.56
Anxiety	17	3.63
Personality disorder	15	3.21
Anxiety	11	2.35
Social phobia	11	2.35
Hypothyreosis	10	2.14
Substance use disorder	9	1.92
Hypertension	9	1.92
Co-medication (*n* = 468, incidence > 1.4%)		
Total	165	35.26
Anti-depressants	113	24.15
Neuroleptics	24	5.13
Thyroid medication	16	3.42
Antiepileptic agents	9	1.92
COPD medication	8	1.71
Other neurotropic medications	7	1.50

Co-medication prescribed consisted mainly of anti-depressants (113 patients, 24.2%), neuroleptics (24 patients, 5.1%), and for somatic indications, thyroid hormone replacement led in 3.4% (16 patients). Overall, 165 patients (35.4%) were on co-medication during the course of the study and 102 patients (21.8%) received psycho-social non-pharmacological add-on therapy.

### Dosing

**Fig. 1:**
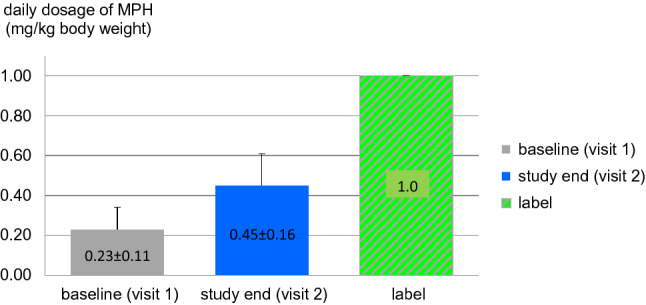
Daily dosage of MPH at baseline (visit 1) and at study end (visit 2). Mean of initial first dosing was 18.4 (±12.7; median 10.0) mg/day, equivalent to 0.23 (±0.11) mg/kg bodyweight. Last dosage at visit 2 was 35.8 (±17.0; median 40.0) mg/day, equivalent to 0.45 (± 0.16) mg/kg bodyweight. The approved label allows 1.0 mg/kg body weight

Overall, data on initial dosing of MPH through last dosage were obtained from 411 patients. Mean of initial first dosing was 18.4 (±12.7; median 10.0) mg/day, equivalent to 0.23 (±0.16) mg/kg bodyweight. Last dosage at visit 2 was 35.8 (±17.0; median 40.0) mg/day, equivalent to 0.45 (±0.21) mg/kg bodyweight. At both visits, minimal dosage was 5.0 mg/day, maximal dosage was 80.0 mg/day (Fig. 1).

### Add-on psycho-social non-pharmacological therapy

In these 468 patients included in the study, 366 patients had no explicit add-on non-pharmacological intervention prescribed. In 102 patients, 122 non-pharmacological interventions were prescribed as add-on therapy with mainly psychotherapy in 30.3% of cases, behavioural therapy in 22.1% and communication therapy in 16.4% and ergotherapy in 9.8% (Table 2).

**Table 2:** Add-on psycho-social non-pharmacological therapy

	Number of patients		
	Absolute	In %	In % adjusted
Intervention			
Pharmacological	366	78.2	
Add-on non-pharmacological	102	21.8	100.0
Non-pharmacological therapy			
Psychotherapy	37	7.9	36.3
Behavioral therapy	27	5.8	26.5
Communication therapy	20	4.3	19.6
Ergotherapy	12	2.6	11.8
Sports	6	1.3	5.9
Social therapy	4	0.9	3.9
Coaching	3	0.6	2.9
Relaxation	3	0.6	2.9
Family therapy	2	0.4	2.0
Day structuring	2	0.4	2.0
Drug counselling service	1	0.2	1.0
ADHD training	1	0.2	1.0
Drug addiction treatment	1	0.2	1.0
Group therapy	1	0.2	1.0
Day care	1	0.2	1.0
Professional re-integration	1	0.2	1.0

### Efficacy assessed by physician and patient

**Fig. 2:**
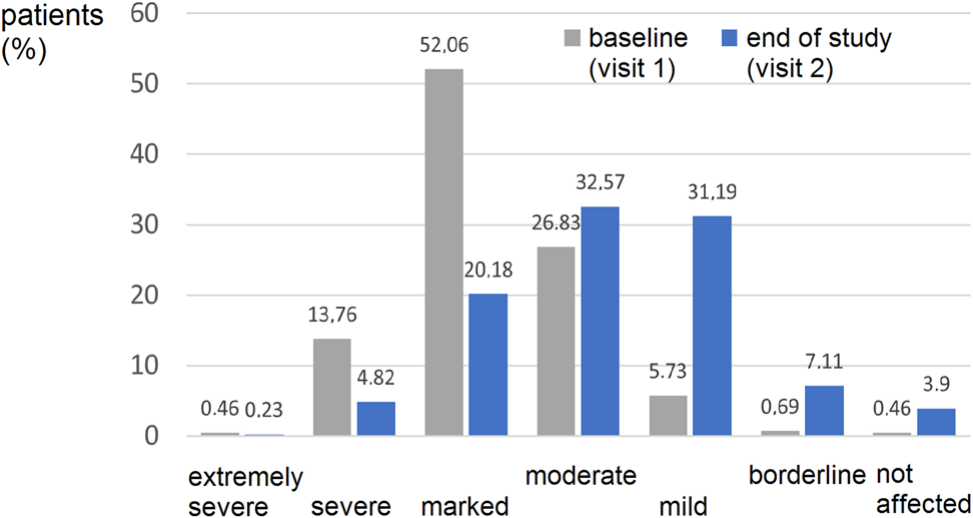
Extent of disorder according to CGI severity scale. Patients were classified into seven categories (not affected to extremely severe) at the beginning (visit 1) and the end of the study (visit 2). Percentage of patients is plotted. Physicians’ assessment showed a significant improvement from baseline to study end

At visit 1, severity of disorder was classified by treating physician as “very severe” or “severe” according to CGI in 62 patients (14.2%), “marked” in 227 patients (52.1%), “moderate” in 117 patients (26.8%), and “light” in 25 patients (5.7%) (Fig. 2). At the end of the observation at visit 2, the portion of (very) severely affected patients was reduced by 64%, and by 61% in markedly affected patients. Overall, the severity of disorder was significantly improved over the course of the study.

**Fig. 3:**
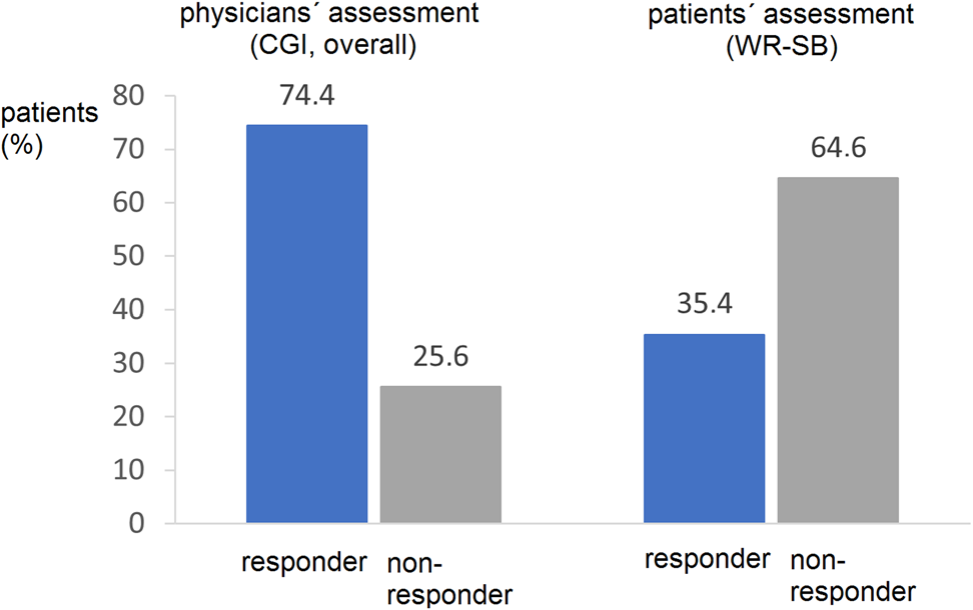
Responder rates according to physicians’ and patients’ assessment. Plotted is the percentage of patients (in %) who are classified as responders according to physicians’ assessment (by means of CGI) or patients’ assessment (by means of WR-SB). Physicians reported a higher portion of patients as responders in comparison to patients (75% vs. 35%)

According to physicians’ assessments based on CGI-I, 74.5% of patients (318 patients) were classified as responders, of which 83 patients (19.4%) were in “much better” and 235 patients (55.0%) in a “clearly better” state (Fig. 3). Slight improvement was registered in 82 patients (19.2%), no change was seen in 26 patients (6.1%), and one patient (0.23%) deteriorated.

Therapeutic efficacy (CGI-efficacy index) was assessed “very good” in 171 patients (40.9%) with complete or nearly complete remission of all symptoms. It was reported that 176 patients (42.1%) revealed significant improvement with partial remission of symptoms, a slight improvement requiring further treatment was seen in 60 patients (14.3%), and 11 patients (2.6%) were regarded as “unchanged” or “deteriorated” (Table 3).

**Table 3:** Efficacy index

Therapeutic efficacy	Adverse events									
	None		Irrelevant		Relevant		Dominating		Total	
	*n*	%	*N*	%	*n*	%	*n*	%	*n*	%
Excellent	156	37.32	14	3.35	1	0.24	0	0	171	40.91
Moderate	157	37.56	14	3.35	4	0.96	1	0.24	176	42.11
Slight	52	12.44	6	1.44	1	0.24	1	0.24	60	14.35
Unchanged, worse	8	1.91	0	0	3	0.72	0	0	11	2.63
Total	373	89.23	34	8.13	9	2.15	2	0.48	418	100

Depicted is therapeutic efficacy (very good, moderate, slight, and unchanged/worsened) in relation to adverse effects (none, irrelevant, relevant, and dominating) in absolute and relative patient numbers (4)

**Fig. 4:**
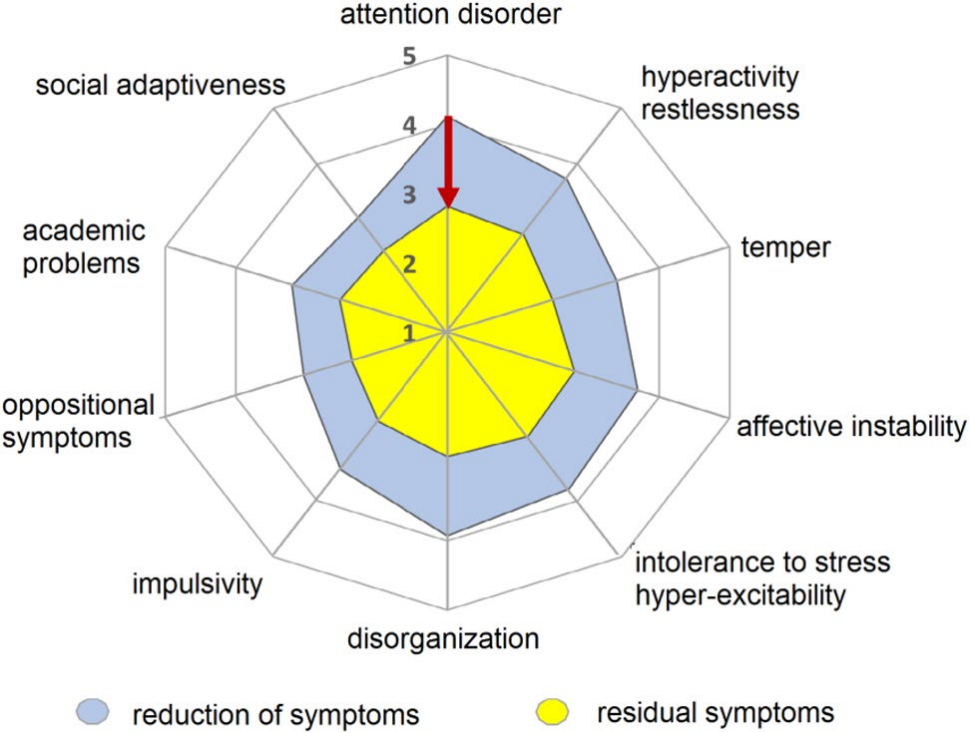
Reduction of symptoms under treatment with methylphenidate according to patients’ assessment (WR-SB). Extent of symptoms classified into ten criteria was documented at the beginning of the study (visit 1) depicted in blue, and at the end of the study (visit 2) as remaining residual symptoms (depicted in red) by means of a 5-step Likert scale (“1 = does not apply to me” to 5 (“applies to me very well”). The graph represents the mean values. In summary, there is a significant improvement of symptoms and their examined sub-scores and an improvement in the intensity and extent of symptoms in adults with ADHD treated with methylphenidate

Patients’ self-assessments based on WR-SB reported a significant improvement (*p* < 0.001) in the underlying sub-scorings (Fig. 4). The total score WR-SB had initially 203.4 (±37.6) points at visit 1 and improved under MPH medication by 50.1 (±40.3) points down to 153.4 (±40.2) points at visit 2 translating into a mean relative reduction of 23.5% and an improvement of symptom severity in adult patients.

According to patients’ assessments, 35.4% of patients (146 patients) were classified as responders (Fig. 3). Multivariate analysis identified severity of disorder at the beginning of study as the main factor for the extent of symptom reduction. In consequence, the number of patients classifiable as responders was much higher in patients with severe or very severe symptoms (responder rate 50.8%). The most obvious reductions were observed with item attention disorder (−30.0%), disorganization (−26.6%), hyperactivity (−23.3%), and poor stress resilience or hyper-excitability (−23.0%). The total number of patients with a much better or better state of health was assessed in 318 patients (74.5%). These patients were matching the definition of 30% improvement. In a multivariate analysis, the relation between potential demographic factors or patient characteristics such as sex, age, severity of disorder, BMI and response were analysed, but revealed no statistical significance (*p* = 0.3265, likelihood ratio test). The co-morbidity of depression showed a slight improved response (*p* = 0.0164). Highest impact on response rate had dosage of stimulants (*p* = 0.0012).

### Safety—adverse events

In 417 patients (89.1%), no adverse events were reported at all. In 51 patients (10.9%), a total of 100 adverse events was documented, i.e. one to two events per patient. Most prevalent was reduced appetite and headache (eight patients each, 1.7%), excitability (five patients, 1.1%), tiredness, and nausea (four patients each, 0.9%) (Table 4). In one patient (0.23%), a severe event (relapse of polytoxicomania) was documented; however, it was classified as unlikely related to MPH.

**Table 4:** Adverse events

	Number of		
	AE	Patients	in %
No AE	0	417	89.1
AE	100	51	10.9
non-severe AE	99	50	98.0
severe AE	1	1	1.96
Death	0	0	0
Type of AE (MedDRA 17.0) *n* = 468. incidence > 0.3%			
Appetite reduced		8	1.71
Headache		8	1.71
Restlessness		5	1.07
Tiredness		4	0.85
Nausea		4	0.85
Diarrhea		3	0.64
Hypertension		3	0.64
Palpitations		3	0.64
Dizziness		3	0.64
Dyssomnia		2	0.43
Heart rate increase		2	0.43
Hyperhidrosis		2	0.43
Dry mouth		2	0.43
Abdominal pain		2	0.43
Tachycardia		2	0.43

Depicted are number, type, and frequency of reported adverse events in absolute and relative frequencies

At visit 1, blood pressure measurements (JNC7 scheme, de Zwaan et al. 2012) were within normal range in 68 patients (17.4%), were pre-hypertensive in 240 patients (61.5%), a hypertension stage 1 or 2 in 82 patients (21.0%). Mean measurement was 124.3/78.9 (±12.1/9.5) mmHg. An increase of 1.1/0.5 (±10.2/9.3) mmHg at the end of the study was regarded clinically insignificant. In analogy, the small change in heart rate from 73.2 (±9.6) beats per minute at visit 1 to 75.3 (±9.9) beats per minute at visit 2 and the reduction of BMI from 25.1 (±4.7) kg/m^2^ to 25.0 (±5.5) kg/m^2^ was also not clinically relevant. Only, a major portion of patients expressed being less hungry. Change in appetite was reported in 10 of 468 patients, 8 patients had a decrease in appetite, 1 patient experienced increased appetite, 1 patient was reported change in appetite. Of interest, decrease in appetite was only reported in patients diagnosed with the inattentive (*n* = 7) or the combined subtype (*n* = 1), but not in the hyperactive-impulsive subtype. Overall, MPH proved to be an effective and safe medication in the treatment of adults with ADHD.

## Discussion

ADHD in adulthood is frequently associated with additional psychiatric disorders like depression and anxiety. If untreated, it leads to a higher risk of substance abuse and problems in social and professional life (Erskine et al. 2016). In comparison to children, the therapeutic options for adults are limited. In Germany, Medikinet® adult was approved in April 2011 as the first medication approved for pharmacotherapy of adult ADHD (Biederman et al. 2006). In the meantime, further methylphenidate preparations are available as well as noradrenalin re-uptake inhibitor atomoxetine.

The efficacy and safety of MPH in adult ADHD patients were shown in three randomized, double-blind, placebo-controlled trials, in EMMA (Rösler et al. 2009) and QUMEA (Retz et al. 2015) and COMPAS (Philipsen et al. 2015; Lam et al. 2019). While in these controlled studies, patients with comorbid psychiatric disorders or co-medication were mainly excluded, our observational study included such patients with psychiatric disorders and co-medication of multiple psycho-pharmacological agents. More than half of the study population (52.6%) suffered from co-morbidities associated with ADHD and more than a third of patients were on co-medication (35.3%). Our sample of patients included in our cohort was recruited from all over Germany: an unusually high number of centers (*n* = 126) was recruited by announcing to all adult psychiatric offices registered in the national society of psychiatry with an interest in ADHD (*n* = 1200) and asking to participate in the trial. The aim was to provide a representative sample of patients recruited in urban and rural areas and avoiding center effects with potential differences in diagnostic and treatment approaches. Thus, our sample had no selection bias and should perfectly represent the usual clientele of adult ADHD patients treated with MPH under real-life conditions.

Intake of MPH has shown no clinically relevant impact on blood pressure, heart rate, or body weight. The observed changes were minimal and clinically insignificant comparable to findings in RCTs (Rösler et al. 2009; Retz et al. 2012). With regard to adverse events, 99 of 100 findings collected during the study period were classified as non-severe and already known and listed in the label (Medice 2017). Only six patients discontinued due to adverse events. In comparison, in QUMEA (Retz et al. 2015), 151 adverse events were registered in 55 patients and in EMMA (Rösler et al. 2009) with 363 patients included 31 patients had to discontinue due to adverse events. In light of these findings, the number and portion of adverse events are relatively low in the study population of this observational study and medication with MPH under routine conditions can be regarded well tolerated and safe.

A recent study addressing prescription patterns of ADHD medications revealed that MPH was the number one medication among all age groups in line with current guidelines and official labels (Bachmann et al. 2017). Also other studies confirm the positive effect of MPH pharmacotherapy on various parameters: MPH reduces reactivity in the amygdala which translates into a positive effect in controlling emotional dysregulation, being a part of the limbic system which plays a relevant role in emotional control (Bouffard et al. 2003). MPH also leads to cognitive enhancement but does not completely normalize but supports psychotherapeutic measures (Fuermaier et al. 2017). In addition, MPH significantly reduces ADHD symptoms and improves everyday functioning and health-related aspects for quality of life (Rösler et al. 2013).

In addition, this study focused on changes in ADHD symptoms and social aspects related to MPH medication. According to physicians’ CGI assessment in 74.5% of cases, CGI improved very much and much under MPH medication which was even higher than in the RCTs (50% in QUMEA, 61% in EMMA). Therapeutic efficacy in relation to adverse events was reported as rated very good in 40.9% of patients at the end of the study, lower than the 60.1% cases reported in EMMA (Rösler et al. 2009). Still, 89% of patients continued MPH medication after the official study end, which clearly indicates a positive benefit-risk assessment and adverse effects not preventing continuation.

Patients also had to undertake self-ratings assessing changes pre- and post- treatment, including social factors and interpersonal problems with colleagues, employers, children and partners. In other words, associated aspects are regarded of high relevance in everyday functioning and quality of life (Harpin 2005; Kooij et al. 2004).

In the observational study, aspects of social adoption were markedly improved under MPH medication. Patients reported a 23.5% improvement in burden of symptoms. 35.4% of patients classified themselves as treatment responders in their self-assessment lower than physicians’ assessments; such discrepancy is generally known from everyday practice and has been reported in scientific literature. Of interest, a recent study revealed arousal regulation as a relevant predictor for stimulant therapy (Strauß et al. 2019). In our study, dosage of methylphenidate correlated positively with response rate. Of notice, daily dosage with 35.8 ± 17 mg MPH was relatively low in our study population. Given the positive dose–effect correlation for MPH (Spencer et al. 1995; our multivariate analysis), we see that therapeutic potential is not fully exploited in daily routine. This finding is of relevance: in randomized controlled trials which have frequently shown good treatment efficacy and good safety profile, the dosing is usually determined by a rigid study protocol. However, in real life such as seen in this observational study, dosing is rather low and matches with only moderate self-rated improvement (Fig. 3). This could be an explanation that the number of prescriptions for ADHD is rather low among adults as underlined by relevant indicators. In adults, diagnostic prevalence is 0.1% and a treatment prevalence is 0.05% with 570 thousand units prescribed with a mean dosage of 0.45 mg/kg BW (IQVIA 2019; Insight health 2019; Thome et al. 2019). In contrast, in children and adolescents, the reported diagnostic prevalence is 4.0% and a treatment prevalence exceeds 2.5% with 1583 thousand units prescribed with a mean dosage of 0.80 mg/kg BW (IQVIA 2019; Insight health 2019). Besides under-dosing, other explanations could be the insufficient level of prescribed non-pharmacological add-on therapy as only 21.8% received such multi-modal treatment or the rather new diagnostic entity and treatment options available for which continuous medical education is needed (Thome et al. 2019). Alternatively, from a different perspective, a reduced burden of disease in progressing age experienced by the patient or better coping strategies, and overall the still limited access to expert therapists as addressed in the recent RAABE study (Thome et al. 2019).

Overall, this observational study is limited by its non-interventional design and thus does not allow the evaluation of cause-effect relations. Nevertheless, the high number of patients included the study represents a comprehensive cross-sectional view in everyday clinical routine with significant findings.

## Conclusion

Severity of ADHD is improved by MPH pharmacotherapy in routine clinical practice. This non-interventional study confirms the positive efficacy and safety profile of MPH for treating adults with ADHD in routine practice as already reported from RCTs (EMMA (Rösler et al. 2009) and QUMEA (Retz et al. 2012) and COMPAS (Philipsen et al. 2015; Lam et al. 2019)). Moreover, the study revealed positive effects on everyday functioning when treated with MPH. Important findings such as relatively low response rate reported in the patient self-assessment and markedly low daily dosage of 40 mg/day indicate that dose titration is not fully exploited in routine practice and may lead to sub-optimal individualized treatment results. A more careful dose titration could further improve the positive benefit of MPH seen in routine care.
